# Influence of elicitation procedure and phrasing on health state valuations in experience-based time trade-off tasks among diabetes patients in China

**DOI:** 10.1007/s11136-019-02292-9

**Published:** 2019-09-12

**Authors:** Shuang Hao, Emelie Heintz, Gert Helgesson, Sophie Langenskiöld, Jiaying Chen, Kristina Burström

**Affiliations:** 1 Health Outcomes and Economic Evaluation Research Group, Stockholm Centre for Healthcare Ethics, Department of Learning, Informatics, Management and Ethics, Karolinska Institutet, 171 77 Stockholm, Sweden; 2 QRC KI, Department of Learning, Informatics, Management and Ethics, Karolinska Institutet, Stockholm, Sweden; 3 Stockholm Centre for Healthcare Ethics, Department of Learning, Informatics, Management and Ethics, Karolinska Institutet, Stockholm, Sweden; 4 Department of Medical Sciences, Uppsala University, Uppsala, Sweden; 5 Centre for Health Policy Studies, Nanjing Medical University, Nanjing, China; 6 School of Health Policy and Management, Nanjing Medical University, Nanjing, China; 7 Equity and Health Policy Research Group, Department of Public Health Sciences, Karolinska Institutet, Stockholm, Sweden; 8 Health Care Services, Region Stockholm, Stockholm, Sweden

**Keywords:** China, Experience-based values, Health state valuation, Iteration-based, Open-ended, Time-trade-off (TTO)

## Abstract

**Purpose:**

Open-ended and iteration-based time trade-off (TTO) tasks can both be used for valuation of health states. It has so far not been examined how the elicitation procedure affects the valuation of experience-based health states. The purpose of this study is to investigate the influence of elicitation procedure on experience-based health state values elicited by the TTO method.

**Methods:**

156 Chinese adults with type 2 diabetes participated in face-to-face interviews with an open-ended or an iteration-based TTO task. The association between the type of TTO task and the valuation of health states was investigated through multiple linear regression analyses. A modified open-ended TTO task was also developed (*n* = 33) to test whether different phrasings of open-ended TTO tasks influence TTO values.

**Results:**

Higher TTO values were observed in the original open-ended TTO task compared to the iteration-based task, which indicates that the elicitation procedure influences the valuation of health states. When the modified open-ended task was introduced, the difference between the two elicitation procedures was no longer statistically significant, suggesting that the phrasing and/or visual presentation of the TTO task may influence the valuation of health states.

**Conclusions:**

The choice of elicitation procedure as well as the description of experience-based TTO tasks may influence the valuation of health states. Further research is warranted, also in other cultural contexts, to further explore these findings.

## Introduction

The Quality-Adjusted Life Year (QALY) is a common outcome measure in economic evaluation of health technologies [1]. QALYs combine length of life with health-related quality of life (HRQoL) on a 0 (dead)—1 (full health) scale. In order to get numeric values for health states, respondents are asked to value their own current health state (experience-based values) or to value health states described to them (hypothetical values) using specific valuation methods, for example, the time trade-off (TTO) method [2]. The TTO method is recommended for valuation of health outcomes in economic assessments by several national health technology assessment agencies [3, 4] and has been used to develop value sets for the EQ-5D instrument in the UK, Spain, Germany, China, Japan, Sweden, Denmark, and the Netherlands [5–12].

TTO values are elicited by asking respondents to make a choice between a certain period of time (usually 10 years) in a particular state with less than full health and a shorter period of time in full health [13–15]. There are different elicitation procedures to do this. TTO values can be elicited using an open-ended question in which the respondent is asked to directly state how many years in full health that he or she finds of equal value to the fixed number of years in the state with less than full health (open-ended TTO). Using this procedure is both time and cost saving and makes it possible to collect TTO data in large population-based surveys. Such a version has been used to develop a Swedish experience-based value set for EQ-5D-3L [10], and similar versions have been employed in other studies [16–19]. TTO values can also be elicited using an iteration-based procedure in which the number of years in full health is varied until the respondent is indifferent between the two alternatives (iteration-based TTO). The health state value is calculated by dividing the number of years in full health that makes the respondent indifferent between the two alternatives with the fixed number of years in the state with less than full health. This version has been used in the development of several value sets based on valuation of hypothetical health states for EQ-5D [5–9, 11, 12, 20–23].

If TTO values from studies using different elicitation procedures are to be used interchangeably, the procedure used should have no impact on the valuation of the health states. In economic theory, this assumption is referred to as procedural invariance. However, it is a known problem that evaluation outcomes often depend on the elicitation procedure [24–26]. Previous studies in the area of health that have compared valuations elicited by open-ended tasks with elicitation through binary choices, such as those used in the iteration-based tasks, have shown that results from these tasks differ [27, 28]. They have all proceeded comparing *described* health states. For instance, in one study [27], the respondents were, among other tasks, asked in one elicitation to choose between living 4 years with more severe heart failure and living a fixed number of years (< 4 years) with less severe heart failure (a binary choice)—and in another elicitation to directly provide the number of years with less severe heart failure that was considered equivalent to 4 years with more severe health failure (open-ended task). The results showed that a higher proportion of participants were willing to trade off years in the binary choice than in the open-ended task (57% vs. 26%). In another study [28], the participants were given the task to choose between living 10 years with back pain followed by death and living fewer years in full health followed by death. The iteration-based task showed a slightly lower TTO value (0.76) than the open-ended task (0.80), indicating that participants were less likely to trade off years in the open-ended task. It should be noted that the focus in these and other studies comparing elicitation procedures primarily has been on preference reversals, and not specifically on the significance of the differences in valuation outcomes.

To the best of our knowledge, no studies have compared elicitation tasks for valuation of *experience*-*based* health states. The purpose of this study is to investigate the influence of elicitation procedure (open-ended or iteration-based questions) on experience-based health state values elicited with the TTO method.

## Methods

### Study population and setting

The study was conducted among Chinese adults (18 years and above) diagnosed with type 2 diabetes. Participants were consecutively recruited and allocated to one of two TTO tasks on an alternating basis by letting every second participant respond to an open-ended task and every second participant respond to an iteration-based task. The sample size of 80 participants in each task was decided based on the results of a previous study investigating the difference between the open-ended and the iteration-based TTO valuation tasks, where the respondents valued hypothetical health states [28]. To detect a statistically significant mean difference of 0.1, with a standard deviation (SD) of 0.18 in each group, a minimum of 50 respondents in each groups would be needed (*α* = 0.05 and 1 − *β* = 0.8).

Individual face-to-face interviews were conducted in March 2016 at the departments of endocrinology of the public hospitals Jiangning Hospital and Nanjing 1st Hospital located in Jiangning and Yuhuatai districts, Nanjing City, Jiangsu province, China. In Jiangning Hospital (a district county hospital), most patients came from the local area, whereas the Nanjing 1st Hospital (a city-level hospital) had patients from different areas of Jiangsu province and also other parts of the country. Patients represent mixed socioeconomic compositions.

### Interview questionnaires

Semi-structured interview questionnaires for open-ended and iteration-based TTO tasks were developed and pilot tested in English and then translated into Chinese and further pilot tested. In both interview questionnaires, the interview started by collecting information about the respondents’ age, sex, duration of diabetes, type of treatment, and comorbidities by asking if the respondent has any other disease diagnosed by a doctor. Information about ethnic group, marital status, having children, education, occupation, and additional comments from the respondent were collected at the end of the interview.

First, the respondents were asked to answer a self-rated health (SRH) question framed as “How is your health today? Is it ‘very good’, ‘good’, ‘fair’, ‘bad’ or ‘very bad’?” [29]. Thereafter, each respondent classified their own health status in five dimensions (mobility, self-care, usual activities, pain/discomfort, and anxiety/depression) and five severity levels for each dimension by answering the EQ-5D-5L instrument [30]. The respondents also reported their own overall health status on a visual analogue scale (EQ VAS) where 100 represents best imaginable health state and 0 represents worst imaginable health state. Next, the respondents completed the TTO task. Finally, the level of difficulty in understanding the task was also assessed through this question “How would you rate the difficulty in understanding this task? Would you say ‘very easy’, ‘easy’, ‘neither easy nor difficult’, ‘difficult’ or ‘very difficult’?”

#### The TTO tasks

**Fig. 1:**
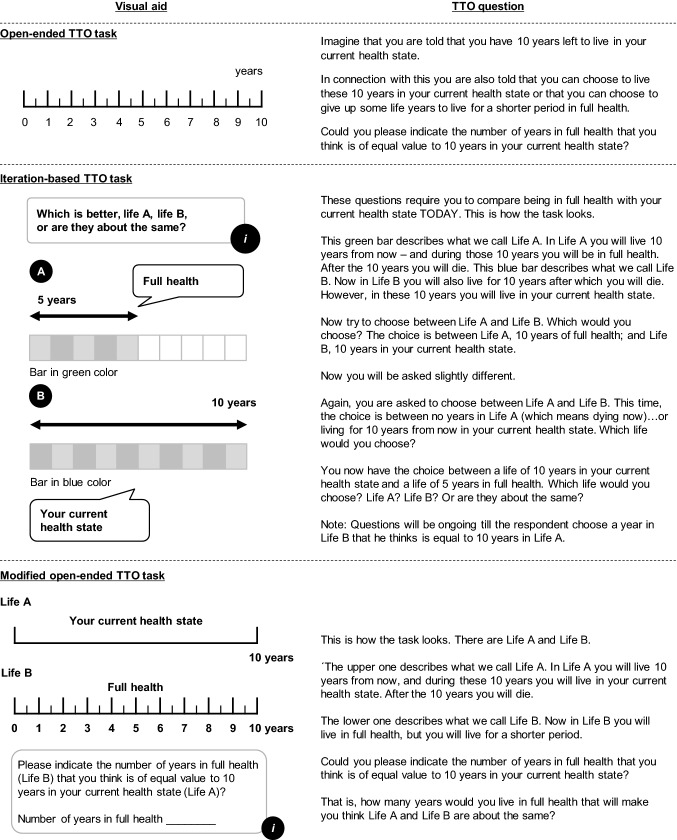
Visual aid and TTO task description of open-ended TTO, iteration-based TTO and modified open-ended TTO tasks

In the open-ended TTO task, the respondent was asked to indicate the number of years in full health (*x*) that would be of equal value to 10 years in his or her current health state (Fig. 1). This question was used in the Swedish experience-based value set for EQ-5D-3L [10]. In the iteration-based TTO task, the respondents were introduced to Life A, in which one will live in full health for a number of years less than 10, and Life B, in which one will live in current health for 10 years. Both lives were said to be followed by death (Fig. 1). The respondents were asked to choose between Life A and B. The years in full health were varied until the respondent indicated that he or she was indifferent between the two lives. The visual aid for the iteration-based task used colors, green for Life A and blue for Life B, and followed the interview script employed in the development of value sets for hypothetical EQ-5D-5L states, but was adapted for valuation of experience-based state [31].

An observation by the interviewers was that some of the respondents to the open-ended task reacted negatively on the suggestion in the time trade-off task that they could choose to “give up” time to live, in exchange for a shorter life in full health. As a response to these reactions, a modified version rephrasing the open-ended question was developed. The modified open-ended TTO task avoided the expressions “10 years left to live” at the very beginning of the task and “give up”, but introduced a comparison between lives A and B, similar to that in the iteration-based task (Fig. 1), which arguably made the modified open-ended task more hypothetical, just like the iteration-based task. The effect of the two different procedures (iteration-based vs open-ended) could therefore be separated from the effect of phrasing and visual presentation. To test this modified version, 37 patients were consecutively recruited from both hospitals after the first 160 interviews were completed.

### Interview procedure

Pilot interviews were conducted by the first author at Karolinska Institutet in Stockholm, in Beijing and at Nanjing Medical University (NMU) in January and February 2016. The first author and two other interviewers from NMU, trained by the first author, conducted the interviews.

Prior to the interview, participants were informed about the purpose of the study, that participation in the study was voluntary and anonymous, that they were free to leave at any time and that their response would have no impact on the health care they receive. Verbal informed consent was obtained from each participant in accordance with the ethical standards of the institutional research committee Nanjing Medical University Ethics Committee.

Data was entered by all interviewers, and the responses to the open-ended questions were translated to English. The data was cross-checked by the first author for quality control. To ensure the confidentiality of personal information, respondents’ names, and contact information were not collected. Ethical approval was obtained by Nanjing Medical University Ethics Committee (2016; # 254).

### Data analysis

Respondents’ characteristics were summarized for the groups responding to the three types of TTO tasks. Independent *t* test for continuous data and Chi square/Fisher’s exact test for categorical data were performed to investigate the comparability of the respondents’ characteristics in the open-ended and iteration-based task groups.

The trade-off was between *x* years in full health and 10 years in the respondent’s current health state. The individual TTO value was calculated by dividing the number of years in full health (*x*) by the number of years in the current health state. Worse health was implied by a lower *x* value (i.e., a shorter period of time in full health was equivalent to 10 years in the present state). In the first analysis, the original open-ended and the iteration-based TTO tasks were included. In an additional analysis, the modified open-ended TTO task was included. This allowed us to compare two effects in relation to the iteration-based procedure: the effect of both procedure and phrasing, and the effect of procedure only. Dummy variables were created to represent the type of TTO task in the regression analyses with the iteration-based task as reference.

We employed multiple linear regression, using ordinary least squares (OLS), to investigate the association between the type of TTO task and the TTO values when controlling for other variables. To obtain the akaike information criterion (AIC) from SPSS we also fitted the same model, using maximum likelihood estimation (MLE), i.e. a generalized linear model with a normal probability distribution and identity link function.

The covariates included in the analyses were age, sex, EQ VAS score, socioeconomic status, and interviewer (Table 1). The first author was selected as reference (Interviewer 1) since this interviewer conducted more pilot interviews and trained the other interviewers. The EQ VAS score was used to control for the respondents’ subjective overall health since it was expected that respondents with more severe health problems would be more likely to trade off years, and have lower TTO values. Socioeconomic status included educational level (below primary school as reference) and occupational status (nonemployed as reference). Outliers were identified by plotting the relationship between TTO values and EQ VAS score, TTO values and SRH levels, as well as EQ VAS score and SRH levels. Adjusted *R*^2^ and AIC were employed to test the goodness-of-fit of the models; the larger the adjusted *R*^2^ and the lower the AIC, the better the goodness-of-fit of the model. Since the residual plot and Breusch–Pagan test both indicated heteroscedastic residuals, White’s robust standard errors were applied to correct for heteroscedasticity [32]. Statistical analyses were carried out by SPSS Statistics version 23.

**Table 1:** Definition of models

Regression model on type of TTO task, EQ VAS score, sociodemographic variables, interviewer	
Model 1	f(type of TTO)
Model 2	f(type of TTO; age)
Model 3	f(type of TTO; age; sex)
Model 4	f(type of TTO age; sex; EQ VAS score)
Model 5	f(type of TTO age; sex; EQ VAS score; educational level)
Model 6	f(type of TTO age; sex; EQ VAS score; educational level; occupational status)
Model 7	f(type of TTO age; sex; EQ VAS score; educational level; occupational status; interviewer)

The level of difficulty in understanding the TTO tasks was investigated by binary logistic regression. Those who answered *difficult* or *very difficult* were coded as having difficulties in understanding the task. The type of TTO task, interviewer, and educational level were selected as explanatory variables. Odds ratios (OR) representing the odds for that the TTO task would be indicated as difficult was examined. The Hosmer and Lemeshow test was employed to check the goodness-of-fit of the model, with nonsignificance of the test representing a good model fit.

## Results

In total, 202 interviews were initiated for the purpose of the study. Of these, nine respondents dropped out because of difficulties in understanding or reluctance to complete the tasks. Of the 193 completed interviews (80 in the open-ended task, 80 in the iteration-based task, and 33 in the modified open-ended task), four outliers were excluded due to apparent inconsistencies between the reported state of their health by SRH, EQ VAS, or TTO values. There were no statistically significant differences between the patients responding to the three types of TTO tasks regarding background characteristics, clinical characteristics, or health outcome measures (Table 2). Almost 50 percent of the respondents from the three groups reported no problems in all dimensions of EQ-5D, and approximately 30 percent rated their health as good or very good. The mean EQ VAS score was similar among the three groups.

**Table 2:** Characteristics of the respondents, by type of TTO task

Variable	Iteration-based (*n* = 80)		Open-ended (*n* = 80)		Modified open-ended (*n* = 33)	
	*n*	%	*n*	%	*n*	%
Mean age in years (SD)	59. 9 (11.3)		56.2 (13.0)		55.9 (10.4)	
Age groups						
27–44 years	6	7.5	15	18.8	5	15.2
45–64 years	45	56.3	43	53.8	23	69.7
65+ years	29	36.3	22	27.5	5	15.2
Sex						
Female	37	46.3	35	43.8	12	36.4
Male	43	53.8	45	56.3	21	63.6
Educational level						
Below primary school	13	16.3	12	15.0	8	24.2
Primary school	17	21.3	11	13.8	7	21.2
Middle school	22	27.5	21	26.3	9	27.3
High school	22	27.5	25	31.3	4	12.1
College and above	6	7.5	11	13.8	5	15.2
Occupational status						
Employed	25	31.3	32	40.0	15	45.5
Retired	35	43.8	33	41.3	11	33.3
Not employed	20	25.0	15	18.8	7	21.2
Clinical characteristics						
Complications-hypertension	36	45.0	25	31.3	14	42.4
Complications-others	22	27.5	20	25.0	9	27.3
Comorbidities other than hypertension	29	36.3	24	30.0	9	27.3
Disease duration ≥ 120 months (months)	31	38.8	26	32.5	12	36.4
Treatment with insulin	58	72.5	64	80.0	29	87.9
Self-rated health (SRH)						
Very good	2	2.5	5	6.3	3	9.1
Good	22	27.5	18	22.5	8	24.2
Fair	51	63.8	44	55.0	19	57.6
Bad	4	5.0	11	13.8	2	6.1
Very bad	1	1.3	2	2.5	1	3.0
Mobility						
Level 1	65	81.3	63	78.8	25	75.8
Level 2	11	13.8	8	10.0	4	12.1
Level 3	2	2.5	4	5.0	–	–
Level 4 and 5	2	2.5	5	6.3	4	12.1
Self-care						
Level 1	76	95.0	75	93.8	30	90.9
Level 2	2	2.5	1	1.3	–	–
Level 3	2	2.5	2	2.5	–	–
Level 4 and 5	–	–	2	2.6	3	9.1
Usual activities						
Level 1	66	82.5	65	81.3	28	84.8
Level 2	11	13.8	9	11.3	1	3.0
Level 3	2	2.5	2	2.5	2	6.1
Level 4 and 5	1	1.3	4	5.0	2	6.1
Pain/discomfort						
Level 1	59	73.8	49	61.3	19	57.6
Level 2	16	20.0	19	23.8	8	24.2
Level 3	5	6.3	6	7.5	5	15.2
Level 4 and 5	–	–	6	7.5	1	3.0
Anxiety/depression						
Level 1	60	75.0	62	77.5	23	69.7
Level 2	16	20.0	14	17.5	7	21.2
Level 3	2	2.5	2	2.5	2	6.1
Level 4	2	2.5	2	2.5	1	3.0
Mean TTO value (SD)	0.70 (0.28)		0.87 (0.18)		0.74 (0.24)	
Mean EQ VAS score (SD)	74.8 (17.7)		77.0 (16.8)		75.1 (21.9)	

Patient characteristics regarding ethnic group (96.3–100% Han), marital status (87.9–96.3% married) and having children (97.5–100%) are not presented in the table due to lack of variation

*SD* standard deviation

### Willingness to trade off years in the different tasks

Calculations of the mean number of years (SD) that the participants were willing to trade off showed 3.04 years (2.76) in the iteration-based task, 1.26 years (1.78) in the open-ended task and 2.61 years (2.37) in the modified open-ended task.

### The open-ended versus the iteration-based TTO task

Without controlling for other variables, the mean TTO value was significantly higher (0.162) for respondents participating in the open-ended TTO task compared to those participating in the iteration-based TTO task (Table 3, Model 1). These results were robust against controlling for other variables in the regression models with coefficients between 0.162 and 0.173 (Table 3, Models 1–7). There were no statistically significant effects on age, sex and interviewer. When controlling for EQ VAS score, a higher EQ VAS score was associated with a significantly lower willingness to trade off years in the TTO task (Table 3, Model 4). When controlling for clinical variables, no statistically significant effects were found (data not shown). There were no systematic differences between educational groups in TTO values. However, those with primary school had significantly higher TTO values compared to those with below primary school as their highest educational level (Table 3, Models 5 and 6). Those employed had statistically significantly lower TTO values than those not employed (Table 3, Model 6). Age, sex, EQ VAS score, socioeconomic status, and interviewer affected the main effect coefficient modestly and increased the *R*^2^ from 0.116 to 0.186, while AIC dropped from − 27.145 to − 28.651, indicating a better goodness-of-fit of the model.

**Table 3:** Multiple linear regression analyses on TTO values, by type of TTO task, EQ VAS score, sociodemographic variables, and interviewer

Variable	Model 1		Model 2		Model 3		Model 4		Model 5		Model 6		Model 7	
	Estimate	*p* value	Estimate	*p* value	Estimate	*p* value	Estimate	*p* value	Estimate	*p* value	Estimate	*p* value	Estimate	*p* value
Intercept	0.717	0.000*	0.666	0.000*	0.676	0.000*	0.403	0.000*	0.334	0.003*	0.368	0.001*	0.297	0.014*
Type of TTO task^a^	0.162	0.000*	0.168	0.000*	0.168	0.000*	0.163	0.000*	0.168	0.000*	0.173	0.000*	0.169	0.000*
Age group^b^														
45–64 years			0.061	0.249	0.057	0.302	0.045	0.402	0.034	0.526	0.046	0.408	0.039	0.481
65+ years			0.044	0.443	0.040	0.492	0.065	0.257	0.066	0.266	0.089	0.190	0.072	0.304
Sex^c^					− 0.012	0.748	− 0.005	0.881	− 0.009	0.803	0.002	0.960	0.002	0.948
EQ VAS score							0.004	0.001*	0.004	0.001*	0.004	0.001*	0.004	0.000*
Educational level^d^														
Primary school									0.131	0.025*	0.150	0.010*	0.148	0.011*
Middle school									0.069	0.201	0.101	0.069	0.112	0.044*
High school									0.066	0.241	0.118	0.048	0.119	0.046*
College and above									0.031	0.665	0.071	0.332	0.078	0.292
Occupational status^e^														
Employed											− 0.105	0.031*	− 0.078	0.127
Retired											− 0.088	0.094	− 0.086	0.102
Interviewer^f^														
Interviewer 2													0.067	0.115
Interviewer 3													0.061	0.148
Observation	156		156		156		156		156		156		156	
AIC	− 27.145		− 24.492		− 22.595		− 31.243		− 28.754		− 29.607		− 28.651	
Adjusted *R*^2^	0.116		0.112		0.107		0.160		0.167		0.181		0.186	
*R*^2^	0.122		0.129		0.130		0.187		0.215		0.239		0.254	

*TTO* time trade-off, *AIC* akaike information criterion

*p < 0.05

^a^Reference group: iteration-based TTO task

^b^Reference group: 27–44 years

^c^Reference group: female

^d^Reference group: below primary school

^e^Reference group: nonemployed

^f^Reference group: interviewer 1

### The modified open-ended versus the iteration-based TTO task

With and without controlling for other variables, there was no statistically significant difference in the TTO value between the respondents to the modified open-ended and the iteration-based TTO tasks (Table 4, Models 1–7). However, there was a significant difference in the TTO value between the respondents to the original open-ended TTO task and to the modified open-ended TTO task (0.113 higher TTO values with the original TTO task compared to the modified task in the full model; data not shown). The effect of controlling for other variables in the models was similar to the effect in the first analysis.

**Table 4:** Multiple linear regression analyses on TTO values (incl. modified open-ended TTO task) by type of TTO task, EQ VAS score, sociodemographic variables, and interviewer

Variable	Model 1		Model 2		Model 3		Model 4		Model 5		Model 6		Model 7	
	Estimate	*p* value	Estimate	*p* value	Estimate	*p* value	Estimate	*p* value	Estimate	*p* value	Estimate	*p* value	Estimate	*p* value
Intercept	0.739	0.000*	0.713	0.000*	0.719	0.000*	0.385	0.000*	0.318	0.000*	0.358	0.000*	0.300	0.004*
Type of TTO task^a^														
Open-ended	0.162	0.000*	0.162	0.000*	0.162	0.000*	0.157	0.000*	0.162	0.000*	0.167	0.000*	0.165	0.000*
Modified open-ended	0.023	0.619	0.019	0.688	0.019	0.677	0.027	0.534	0.039	0.370	0.048	0.270	0.052	0.227
Age group^b^														
45–64 years			0.014	0.772	0.011	0.819	0.010	0.841	− 0.003	0.948	0.007	0.893	0.006	0.900
65+ years			− 0.012	0.827	− 0.014	0.790	0.030	0.561	0.030	0.584	0.050	0.424	0.042	0.512
Sex^c^					− 0.007	0.823	− 0.001	0.968	− 0.015	0.659	0.000	0.993	0.003	0.937
EQ VAS score							0.004	0.000*	0.004	0.000*	0.004	0.000*	0.004	0.000*
Educational level^d^														
Primary school									0.142	0.005	0.170	0.001*	0.173	0.001*
Middle school									0.069	0.148	0.103	0.035*	0.111	0.022*
High school									0.084	0.097	0.136	0.012*	0.139	0.010*
College and above									0.043	0.500	0.087	0.181	0.095	0.143
Occupational status^e^														
Employed											− 0.108	0.016*	− 0.086	0.067
Retired											− 0.092	0.053	− 0.086	0.069
Interviewer^f^														
Interviewer 2													0.058	0.126
Interviewer 3													0.041	0.283
Observation	189		189		189		189		189		189		189	
AIC	− 27.226		− 23.750		− 21.800		− 39.853		− 40.084		− 42.255		− 40.689	
Adjusted *R*^2^	0.099		0.092		0.087		0.174		0.193		0.211		0.212	
*R*^2^	0.108		0.111		0.111		0.201		0.236		0.261		0.271	

*TTO* time trade-off, *AIC* akaike information criterion

*p < 0.05

^a^Reference group: iteration-based TTO task

^b^Reference group: 27–44 years

^c^Reference group: female

^d^Reference group: below primary school

^e^Reference group: nonemployed

^f^Reference group: interviewer 1

### Difficulty in understanding TTO tasks

In total, about 15% (*n* = 193) of the respondents indicated that the TTO tasks were difficult to understand. However, there were no statistically significant differences regarding the odds of reporting the task as difficult between the groups responding to the different TTO tasks (Table 5). When comparing respondents with different educational levels, those with the highest education level were significantly less likely to report difficulties with understanding the task compared to those with the lowest educational level (in Model 1 with the open-ended and iteration-based tasks). Those who were interviewed by Interviewer 3 were significantly less likely to report difficulties (Model 1). When entering the modified open-ended task, there was no difference between the interviewers (Model 2). In these analyses regarding the difficulties in understanding, the four outliers were included.

**Table 5:** Binary logistic regression investigating analyses on difficulty in understanding TTO tasks by type of TTO task, interviewer, and educational level

Model 1 (*n* = 160)					Model 2 (*n* = 193)				
Variable	OR	*p* value	95% Wald CI for OR		Variable	OR	*p* value	95% Wald CI for OR	
			Lower	Upper				Lower	Upper
Threshold	0.523	0.299			Threshold	0.521	0.270		
Type of TTO task^a^					Type of TTO task^a^				
Open-ended	2.350	0.090	0.875	6.314	Open-ended	2.390	0.084	0.889	6.426
					Modified open-ended	1.693	0.391	0.508	5.642
Interviewer^b^					Interviewer^b^				
Interviewer 2	1.142	0.805	0.398	3.281	Interviewer 2	1.204	0.706	0.458	3.167
Interviewer 3	0.177	0.040*	0.034	0.921	Interviewer 3	0.283	0.053	0.079	1.018
Educational level^c^					Educational level^c^				
Primary school	0.430	0.215	0.113	1.633	Primary school	0.409	0.126	0.130	1.285
Middle school	0.203	0.019*	0.054	0.767	Middle school	0.178	0.004*	0.055	0.578
High school and above	0.099	0.001*	0.025	0.391	High school and above	0.077	0.000*	0.021	0.284
Hosmer and Lemeshow test	0.867				Hosmer and Lemeshow test	0.678			

*OR* odds ratio, *CI* confidence interval

*p < 0.05

^a^Reference group: iteration-based TTO task

^b^Reference group: interviewer 1

^c^Reference group: primary school and below

## Discussion

To the best of the authors’ knowledge, this is the first study to investigate the influence of elicitation procedure—open-ended and iteration-based—on experience-based health state values elicited with the TTO method. In this study, ‘experienced-based value’ refers to the value of the individual’s currently experienced health state. However, ‘experience’ may have other connotations, such as past experience (has had a disease) or vicarious experience (knowing someone having a disease) [33].

Our results indicate that there is an association between the elicitation procedure and the valuation of health states—which is in line with previous studies [24–28]—among patients with type 2 diabetes in face-to-face interviews in China. The use of an open-ended TTO task tends to lead to a higher valuation of the health state than the iteration-based approach. In other words, respondents tend to trade off years to a greater extent when using iteration-based TTO. The modified open-ended TTO task was introduced and tested to avoid the negative perception among some of the respondents regarding the wording “10 years left to live” and “give up” in the original open-ended TTO task. With this rephrasing, there was no longer a statistically significant difference between the open-ended and the iteration-based TTO elicitation procedures. Instead there was a significant difference between the two versions of the open-ended TTO task. The larger difference between the two versions of the open-ended task, compared to that between the modified open-ended tasks and the iteration-based task, suggests that the phrasing of the task used in the elicitation procedure might influence the valuation of health states more than the elicitation procedure itself.

### Explanations

There are several potential explanations for our results. One possible explanation for the higher values elicited from the open-ended TTO task is loss aversion, which refers to the concept of people giving more weight to losses than to gains [27, 34, 35] and thus being more reluctant to give up life years in the TTO task than they are willing to gain higher quality of life for the lesser number of years. The effect of loss aversion has been suggested to be stronger in the open-ended task than in the iteration-based task, since the open-ended task puts more emphasis on the fact that something has to be given up [28]. Another possible explanation for the differences in values brought up in the literature refers to the phenomenon of scale compatibility, the idea being that responders are partly steered by what is the dominating scale. Compared to the iteration-based task, respondents in the open-ended TTO task have been seen to give more weight to the attribute of time than the potential improvement of health, since the response scale is more prominent in the open-ended task [28].

Common for both these explanations of the difference in outcome when eliciting TTO tasks in different ways is that they refer to certain differences in how the tasks are *described*. While there are no inherent differences regarding the core valuation of the two elicitation procedures of the TTO task—valuing a state of less than full health compared to one with full health, where the valuation is expressed in terms of a time trade-off—there may nevertheless be differences in how this has been described that might influence the valuations. Differences may concern how the task is introduced and described in words, but also how it is visually presented. Visual presentation, in turn, may vary both in terms of how scales are stressed (loss of life, gain in health) and how the very comparison is graphically illustrated (including variations in use of color). For instance, in our study the original open-ended TTO task was visualized by a single scale while the iteration-based TTO task was presented by the use of two bars, presented as Life A and Life B, marked with different numbers of years (Fig. 1). The iteration-based task started with the bar representing years in full health at 5 years (Life A), and the one representing the respondent’s present health at 10 years (Life B). Perhaps it comes more naturally to think of the single scale as representing one’s own life, while the two bars can more easily be perceived as hypothetical alternatives, thus somewhat shifting the choice from being about one’s own life to being about two hypothetical lives. As the modified open-ended TTO task was also described with two bars, even this task might have been perceived as hypothetical, which in turn might explain the similarity between this and the iteration-based task. Such perception can, of course, be strengthened or weakened depending on how the choice situation is described in words. We suggest that differences in how the tasks were phrased and visually presented in our study may have influenced the results [36].

An obvious difference between open-ended and iteration-based TTO tasks relates to the repetitiveness of the iteration-based approach. This may have two effects: on one hand, the iteration may provide a means to step by step target a valuation that accurately reflects the values of the respondents. On the other hand, the respondents might lose some of their resistance to the idea of trading off time, or at least, lost or not, behave in greater accordance with the conditions of the task: to trade off time as an expression of their valuation of the health state. It may also be the case that the open-ended TTO task better accommodates, and accounts for, a genuinely felt resistance to the very conditions of the task. Yet there are other differences between the tasks since the iteration-based task emphasizes the respondent’s current health state TODAY, whereas in the open-ended tasks, TODAY is not mentioned. This means that slightly different recall periods might be considered in the different tasks—say, the present day compared to this week or this month. However, all respondents were at the beginning of the interviews instructed to think about their health TODAY as EQ-5D-5L and EQ VAS have this recall period, so with this background the discrepancy between the two elicitation procedures is reduced. We have used formulations of questions currently used in other studies, and we cannot know the impact on this discrepancy in the present study [10, 31].

### Strengths and limitations

A strength of this study is that the tasks have been administrated through face-to-face interviews: it provided the possibility for the interviewers to get a better understanding of to what extent the respondents understood the question. It was also possible to identify the potential impact of the wording of the open-ended TTO tasks. However, face-to-face interviews may also present some potential problems related to the presence of an interviewer. For example, it was the impression of the interviewers that some respondents did not want to admit to the interviewer that they had health problems even though this was apparent in their responses to the background questions. This is consistent with previous studies showing that respondents report better health during an interview than in other administration modes [37, 38].

Another strength of the study is that it contained all background variables, except for religious beliefs, suggested by van Nooten et al. [39] for TTO studies. However, the background variables ethnic group, marital status, and having children could not be analyzed due to the lack of variation within these variables. There were no statistically significant differences in background variables between participants in the different tasks, which might be taken to suggest that the study design did not introduce any bias. However, even though we have adjusted for several possible confounders in the regression analysis, there could be other differences between the groups that may have influenced the results.

The interviews were conducted in the wards, which meant that interferences due to routine follow-ups and the visits of relatives could not be avoided. Interferences by relatives wanting to help out were avoided by asking the relatives not to interfere during the interview. Asking questions about diseases before the TTO task might influence the answers. In this study, we asked for how long the respondent had been diagnosed with diabetes and if the respondent had any other disease diagnosed by the doctor as well as the SRH question. We cannot say whether this influenced the willingness to trade off time. Another limitation is that the 37 respondents answering the modified open-ended task were not randomly selected.

In this study, we showed that how the TTO task is phrased and visually presented affects the valuations. However, we made changes both in the phrasing and in the visual presentation for the group that responded to the modified open-ended task, which leaves us no way of telling which factor had the greatest influence on the outcome compared to the original open-ended TTO task. Future studies should investigate this by separating the two modifications to be able to make comparisons.

Some previous studies show that Chinese people are conservative in reporting poor health-related quality of life [40, 41]. Of relevance to the generalizability of our results, it has been suggested that Chinese people have a tendency to be more grateful for life than other populations [40, 42–44] and more directed at avoiding death (both Taoism and Buddhism are pursuing immortality)—living with poor health is preferred to a good death [42, 45]. Because death, and talking about death, can be seen as taboo in Chinese culture [45], it is possible that Chinese people are more likely to react negatively to the typical way to describe the open-ended TTO question compared to respondents from other cultures. The results can therefore not be simply generalized to other cultures.

In addition, we do not know whether differences similar to the ones found in this face-to-face study would occur in studies using other modes of administration. Further, we cannot tell whether we can generalize from patients with diabetes to other groups of patients.

Generally, the open-ended task has the advantage over the iteration-based as it is less burdensome for respondents, takes less time to complete, can be administrated in postal surveys, and hence costs for data collection will be lower. In the Chinese context, in the aspect of cultural relations to death (trading off years), the modified open-ended task produces as consistent estimates as the iteration-based which is used in the development of value sets for hypothetical EQ-5D-5L states [31]. The iteration-based TTO task has also been shown to be challenging in a nationally representative Chinese population survey used for estimation of a TTO value set for EQ-5D-3L [44].

Further research is needed to explore how the elicitation procedures as well as the phrasing and visual presentation of TTO tasks influence the valuation of experience-based health states in different cultural contexts using different modes of administration.

## Conclusion

The findings of this study show an association between the elicitation procedure and the valuation of health states in experience-based TTO tasks, showing higher TTO values from an open-ended TTO task compared to an iteration-based TTO task. When a modified open-ended task was introduced, the difference between the two elicitation procedures was no longer statistically significant. The results suggest that the description of the open-ended TTO task influences the valuation of health states. Both variations in phrasing and in visual presentation can have this effect. Further research is needed to explore how the elicitation procedures and descriptions of TTO tasks influence the valuation of experience-based health states in different cultural contexts.
